# *C. elegans* Model Identifies Genetic Modifiers of α-Synuclein Inclusion Formation During Aging

**DOI:** 10.1371/journal.pgen.1000027

**Published:** 2008-03-21

**Authors:** Tjakko J. van Ham, Karen L. Thijssen, Rainer Breitling, Robert M. W. Hofstra, Ronald H. A. Plasterk, Ellen A. A. Nollen

**Affiliations:** 1 Department of Genetics, University Medical Centre Groningen and University of Groningen, Groningen, The Netherlands; 2 Groningen Bioinformatics Centre, University of Groningen, Haren, The Netherlands; 3 Hubrecht Laboratory, Netherlands Institute of Developmental Biology, Utrecht, The Netherlands; Stanford University Medical Center, United States of America

## Abstract

Inclusions in the brain containing α-synuclein are the pathological hallmark of Parkinson's disease, but how these inclusions are formed and how this links to disease is poorly understood. We have developed a *C. elegans* model that makes it possible to monitor, in living animals, the formation of α-synuclein inclusions. In worms of old age, inclusions contain aggregated α- synuclein, resembling a critical pathological feature. We used genome-wide RNA interference to identify processes involved in inclusion formation, and identified 80 genes that, when knocked down, resulted in a premature increase in the number of inclusions. Quality control and vesicle-trafficking genes expressed in the ER/Golgi complex and vesicular compartments were overrepresented, indicating a specific role for these processes in α-synuclein inclusion formation. Suppressors include aging-associated genes, such as *sir-2.1/SIRT1* and *lagr-1/LASS2.* Altogether, our data suggest a link between α-synuclein inclusion formation and cellular aging, likely through an endomembrane-related mechanism. The processes and genes identified here present a framework for further study of the disease mechanism and provide candidate susceptibility genes and drug targets for Parkinson's disease and other α-synuclein related disorders.

## Author Summary

Parkinson's disease is the second most common brain disorder of the elderly. It is thought to be caused by environmental and genetic factors. However, little is known about the genes and processes involved. Pathologically, Parkinson's disease is recognized by inclusions in the brain that contain a disease-specific protein: alpha-synuclein. We created a small animal model (*C. elegans*) in which we could follow the formation of alpha-synuclein inclusions in living and aging animals. With a genome-wide RNAi screen we identified 80 genes whose expression influences inclusion formation. These genes include evolutionarily conserved regulators of longevity, suggesting a link between inclusion formation and the molecular mechanism of aging. Our results offer a refined understanding of how Parkinson's disease arises during aging and we identify processes and genes that may underlie an increased susceptibility for the disease, which is important for improving diagnostics and developing strategies for therapeutic intervention.

## Introduction

Sporadic as well as familial Parkinson's disease are characterized by protein inclusions in the brain containing α-synuclein [1]. Similar inclusions are also present in other neurodegenerative diseases, including dementia with Lewy bodies [2]. The α-synuclein gene is causatively related to Parkinson's disease, since mutations in the gene, and duplication or triplication of the α-synuclein locus cause familial forms of Parkinson's disease in humans [3]–[5]. Sporadic Parkinson's disease, seen in 1–4% of the population over 65 years of age, appears to be unrelated to mutations or multiplications of the α-synuclein locus. How α-synuclein inclusions are produced is unknown, but identifying cellular factors and processes involved in the formation of these inclusions may provide some understanding of the molecular cause of Parkinson's disease and of the link between aging and the sporadic form of the disease. To study pathological α-synuclein accumulation, we used a genetic model organism, the nematode *Caenorhabditis elegans*. We chose *C. elegans* for its thoroughly characterized aging properties, its amenability to genome-wide RNAi screening, and its transparency throughout its lifetime, which allows visualization of inclusions in living animals during aging. We expressed human α-synuclein fused to yellow fluorescent protein in the body wall muscle of C. elegans, where it, age-dependently, accumulated into inclusions. In old age these inclusions contained aggregated material, similar to human pathological inclusions. We used a genome-wide RNAi screen to identify genes and cellular processes involved in age-related α-synuclein accumulation in inclusions.

## Results/Discussion

**Figure 1:**
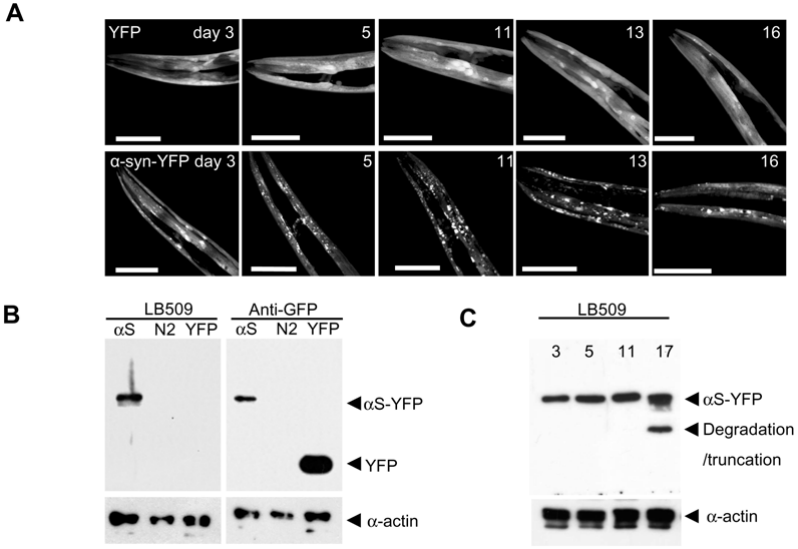
Α-synuclein-YFP in Transgenic Animals Relocalizes to Discrete Inclusions during Aging. (A) Confocal laser scanning images showing α-synuclein-YFP expression in the head region of transgenic *C. elegans* during aging. (B) Immunoblotting analysis of SDS/PAGE separated protein extracts from α-synuclein-YFP, N2 (wt) and YFP animals using α-synuclein (LB509) and YFP (anti-GFP) antibodies. Loading control is α-actin. (C) Immunoblotting analysis of protein extracts from 3-, 5-, 11- and 17-day old α-synuclein-YFP synchronized animals using anti-α- synuclein antibody.

To visually trace expression of α-synuclein, we expressed human α-synuclein fused to yellow fluorescent protein (YFP) in *C. elegans* under control of the *unc-54* promoter, which drives expression to the body wall muscle cells. Muscle expression rather than neuronal expression was chosen for several reasons. The *unc-54* promoter is strong and muscle cells are large, allowing for visual detection of α-synuclein expression and its subcellular localization. Furthermore, RNAi by feeding seems to work more efficiently in muscles than in neurons, which better allows for genome-wide RNAi screening. Finally, muscle expression has been used successfully to model protein-misfolding diseases and to identify modifier genes in previous studies [6]–[8]. The α-synuclein-YFP chimaeric protein is recognized by an antibody specific for human α-synuclein and an antibody for YFP (Figure 1B). YFP fused to human α-synuclein relocates to inclusions (Figure 1A), which are visible as early as day 2 after hatching and increase in number and size during the animals' aging up to late adulthood. As YFP alone remains diffusely localized throughout aging, this indicates that relocation of α-synuclein-YFP into foci is caused by intrinsic properties of the α-synuclein protein.

**Figure 2:**
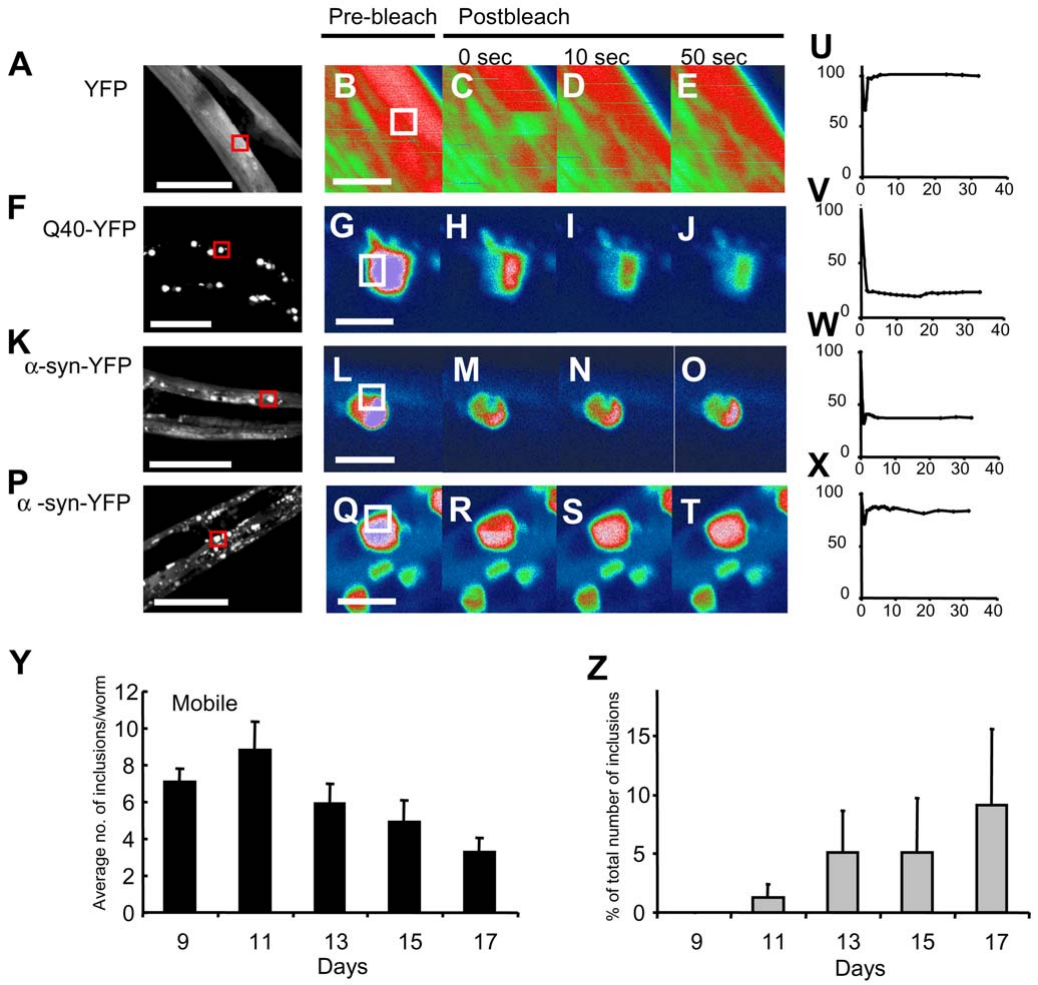
Fluorescent Recovery after Photo Bleaching Reveals α-Synuclein Inclusions Contain Mobile as well as Immobilized Protein Material. (A,F,K,P) Images of YFP, Q40-YFP and α-synuclein-YFP transgenic animals. (B-E,G-J, L-O,Q-T) High magnification images of the area indicated (red box) before photo bleaching and during recovery. (U-X) Graphical representation of fluorescence recovery after photo bleaching in (B-E, G-J, L-O, Q-T). Relative fluorescence intensity (RFI) value on y-axis represents percentage fluorescence corrected for background bleaching. (Y) Average number of inclusions larger than ∼2 µm2 per animal between tip of the nose and pharyngeal bulb during aging (n = 9 for day 11, n = 10 for days 9, 13, 15 and 17). (Z) Percentage of foci containing immobile material during aging. Bar in d-g represents 50 µm (overview) and 5 µm in higher magnification images. Error bars in (Y) indicate standard deviation.

One of the characteristics of late inclusions in the brains of Parkinson's patients is the presence of electron-dense filamentous and granular protein material, which is typical for aggregated protein [9]. To address whether α-synuclein was aggregated within the inclusions in our *C. elegans* model, we measured the mobility of the α-synuclein-YFP chimaera by fluorescence recovery after photo bleaching (FRAP) [10]. We observed two types of inclusions. One type contained mostly mobile material (Figure 2P–2T, 2W; ∼80% fluorescence recovery), whereas the other type contained immobilized material (Figure 2K-2O, 2X; ∼40% fluorescence recovery), similar to Q40- YFP aggregates (Figure 2F-2J, V; ∼30% fluorescence recovery), indicating aggregated protein, a characteristic of α-synuclein deposits in Parkinson's disease.

Notably, there was an increase in the number of “immobile” inclusions relative to “mobile” inclusions during aging (Figure 2Y and 2Z), which appeared unrelated to the expression level of α- synuclein-YFP (Figure 1C). Interestingly, immobile inclusions were not observed before late adulthood, which is consistent with age-related aggregation in Parkinson's disease patients [11]. We noted that the motility of the mobile material contained in inclusions was similar at all ages, suggesting that aggregation was not a gradual process but caused by a sharp transition from mobile to immobile material (data not shown). Taken together, the time-dependent accumulation and immobilization of α-synuclein in inclusions verify the suitability of our α-synuclein-YFP *C. elegans* system as a model for human Parkinson's disease.

**Figure 3:**
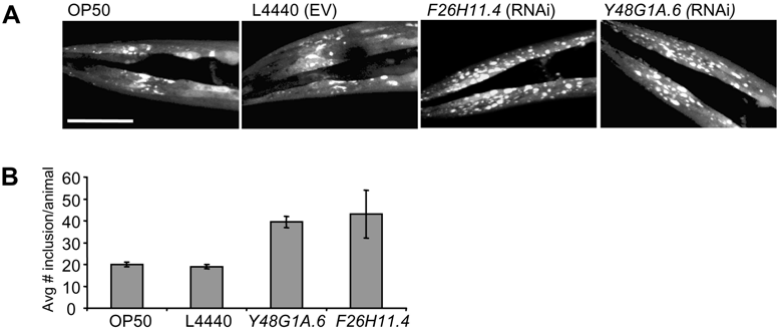
Suppressors of Inclusion Formation Identified by RNAi. (A) Confocal images showing head region of α-synuclein-YFP transgenic animals fed on OP50 bacteria, bacteria containing L4440 (empty vector) and expressing double stranded RNA targeting two representative genes (*F26H11.4* and *Y48G1A.6*) found to increase inclusion formation. Phenotypes of increased inclusion formation were analyzed in liquid culture by observing at least five out of the first ten animals screened to show an increased presence of inclusions compared to wild type. Scale bar represents 50 µm. (B) Quantification of the number of inclusions present in worms as shown in (A) (n = 2).

To identify processes involved in inclusion formation we searched for genes that, when inactivated, increased the amount of inclusions using a genome-wide RNAi screen. Worms were screened by visual inspection using fluorescence microscopy at days 4 and 5 after transfer of synchronized, first stage (L1) larvae to RNAi clones. Clones were scored positive when at least five out of the first ten worms screened showed an increase in the amount of inclusions (Figure 3). Genes for which RNAi results were confirmed in duplicate, in liquid culture and on agar plates, were considered suppressors of inclusion formation.

**Figure 4:**
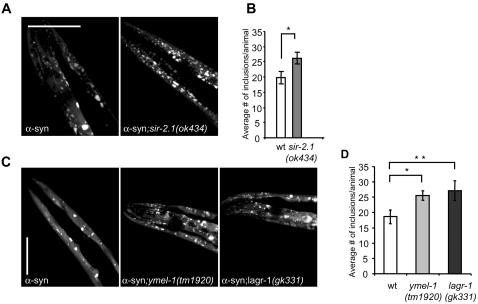
*Sir-2.1*, *ymel-1* and *lagr-1* Are Suppressors of α-Synuclein Inclusion Formation. (A,B) Confocal images showing α-synuclein-YFP transgenic animals and the transgenic strains containing a deletion in the *sir-2.1*gene (*sir-2.1*(*ok434*)) on day 9. (C) Average number of inclusions between tip of the head and pharyngeal bulb of the worm (n = 8). *P≤0.025 (Student's *t* test). (D, E, F) Confocal images showing α-synuclein-YFP transgenic worms and the transgenic strains containing a deletion in the *ymel-1*and *lagr-1* gene (*ymel-1*(*tm1920*) and *lagr-1*(*gk331*)), on day 9. (G) Average number of inclusions between tip and pharyngeal bulb of the worm (n = 8 (wt and *ymel-1*), n = 7 (*lagr-1*)). *P≤0.05, **P≤0.05 (Student's *t* test).

In this screen we found 80 suppressors of inclusion formation (Table 1). Forty-nine of these genes have an established human ortholog (Blast E-value ≤2.5e-5), indicating involvement of these genes in molecular pathways conserved between humans and nematodes (Table S1 online). The effect of RNAi was confirmed in genetic deletion strains for three genes: *sir-2.1*, an NAD+-dependent protein deacetylase, *lagr-1*, a sphingolipid synthase, and *ymel-1*, a mitochondrial protease, which is an ortholog of the human presenilin associated metalloprotease (PAMP) (Figure 4).

**Table 1:** Suppressors of α-Synuclein Inclusion Formation.

Function	Cosmid no.	Gene	Description	Strength
CS	R05H10.6	*cdh-7*	FAT tumor suppressor homolog 3	+
CS	Y54E2A.2		Similar to Ca2+-binding actin-bundling protein (spectrin)	+
DR	E03A3.2	*rcq-5*	DEAD/DEAH box helicase	++
DR	F32D1.10	*mcm-7*	DNA-replication licensing factor	+
DR	W02D9.1	*pri-2*	Eukaryotic-type DNA primase, large subunit	++
ECM	F41F3.4	*col-139*	Structural component of the cuticle, phosphate transport	+
EM	C07A9.8		Bestrophin, anion channel	++
EM	C28H8.11		Tryptophan 2,3-dioxygenase	++
EM	R03E1.2		Renin-precursor, lysosomal ATPase H+ transporter	++
EM	T14F9.1	*vha-15 (phi-52)*	ATPase coupled proton transport, Vacuolar ATP synthase sub	+
EM	Y37H9A.6	*ndx-4*	NUDIX hydrolase	+
ET	B0213.12	*cyp-34A7*	Cytochrome P450 2B6	+
ET	W01B11.2	*sulp-6*	STAS domain, Sulfate transporter family	++
ET	W01B11.6		Thioredoxin, Yeast: required for ER-Golgi transport, stress protection	++
GM	F29F11.2		UDP-glucuronosyltransferase 1–8 precursor	++
GM	K07A3.1	*fbp-1(K07A3.b)*	Fructose-1-6-bisphosphatase	+
LM	C28C12.7	*spp-10*	sphingolipid metabolism/lysosomal	++
LM	F28H1.4		Membrane-associating domain, Plasmolipin	++
LM	F54F11.1		Lipolytic enzyme, putative phospholipase	++
LM	K09H9.6	*lpd-6*	RNA-binding protein required for 60S ribosomal subunit biogenesis	+
LM	T08H10.1		Aldo-keto reductase family 1 member B10, oxido-reductase	+
LM	W03G9.6	*paf-1*	acetylhydrolase/phospholipase A2	++
LM	Y48G9A.10		Carnitine O-palmitoyltransferase I	++
LM	Y6B3B.10	*lagr-1*	LAG1, (dihydro)ceramide synthase, spingholipid synthesis, ER	++
PD	C46F9.3	*math-24*	MATH domain, Ubiquitin carboxyl-terminal hydrolase 7	++
PD	C47B2.1		F-box domain	+
PD	F32H2.7		E3 ubiquitin-protein ligase HECTD1	+
PD	F49B2.6		Peptidase family M1	+
PD	M03C11.5	*ymel-1*	Peptidase M41, FtsH, metallo protease: unfolded mito-proteins	++
PD	R09B3.4	*ubc-12*	Ub-conj. enzyme (E2), NEDD8-conj. enzyme NCE2	+
PD	R151.6		Human Derlin-2, protein degradation in ER	+
PD	T06A4.1		Peptidase M14, carboxypeptidase A, Zinc-metalloprotease	+
PD	Y63D3A.9	*fbxb-93*	F-box	+
PF	F52C12.2		Uncharacterized conserved protein	+
PF	R151.7		Hsp90 protein	++
PS	F42A10.4	*efk-1*	Ca/calmodulin-dep. kinase, Elongation factor 2 kinase	+
PS	F56H6.9		Protein of unknown function, DUF288	+
PS	Y67D8A_370.a	*puf-4*	Translational repression, Splice Isoform 2 of Pumilio homolog 2	++
RSP	C04F5.5	*srab-2*	7TM chemoreceptor, GPCR activity, DNA biding/Zn-finger	+
RSP	C28H8.5		ShTK domain	++
RSP	D2089.1	*rsp-7*	Splicing factor, arginine/serine-rich	++
RSP	F26H11.4		Neurofilament triplet H protein	++
RSP	T12A2.3		Protein AF-9	++
RSP	Y113G7B.18	*mdt-17*	Transcriptional cofactor	+
RSP	Y116A8C.35	*uaf-2*	Splicing factor U2AF 35 kDa subunit	+
RSP	Y48G1A.6	*Y48G1A_53.a*	Polycomb group protein SCM/L(3)MBT	++
SG	C24B9.8	*str-13*	7TM chemoreceptor, GPCR activity	++
SG	F10E9.3		Splice Isoform 1 of Death domain-associated protein 6	++
SG	F12A10.6		Serine/threonine kinase (haspin family)	++
SG	F19H8.2		∼to Rho kinase	+
SG	F21F3.3		Isoprenylcysteine carboxyl methyltransferase family, yeast: ER	++
SG	R09E12.1	*srbc-59*	7TM chemoreceptor, srbc family	++
SG	R52.4		Chemoreceptor	+
SG	T25B9.2		protein phosphatase 1, catalytic subunit, alpha isoform 3	++
SG	T28F2.2		Splice Isoform 1 of COMM domain-containing protein 4	++
SG	W05B5.2		GPCR, membrane	++
SG	Y71G12B.20	*mab-20*	Semaphorin-4G precursor	++
SG	ZK355.1	*ZK355.h*	Tyr-kinase Receptor	++
U	B0238.11		HMG box-containing protein, transcriptional	+
U	C02E7.6		Splice Isoform 1 of AMME syndrome candidate gene 1 protein	++
U	C29F9.1		Unknown	++
U	D1022.5		Dienelactone hydrolase family	+
U	F01G12.5a	*let-2*	Unknown	++
U	F47F6.5		Similarity to C-type lectin	++
U	F54E7.6		Unknown	++
U	F56C4.1		Unknown	+
U	H23L24.e		Unknown	++
U	K01G12.3		HIV TAT specific factor 1	+
U	R09H3.3		Unknown	++
U	R10H10.2	*spe-26*	kelch-like 20	++
U	R11A8.4	*sir-2.1*	NAD-dependent deacetylase sirtuin-1	++
U	T05A10.4		Similarity to cysteine-rich secretory protein 2 precursor	+
U	T06G6.8		Unknown	++
U	XE249		Chondroitin 6-sulfotransferase	++
U	Y19D10A.j		C-type lectin	+
U	ZC334.9	*ins-28*	Insulin-like peptide	++
U	ZK1290.11		Unknown	++
VT	C07G1.5	*hgrs-1 (pqn-9)*	HGF-reg tyr-kinase substrate, membrane trafficking/protein sorting	+
VT	C34C12.2		Role in preribosome assembly or transport/t-snare domain	++
VT	M151.3		Girdin, Yeast: ER-Golgi transport, SNARE assembly	++
VT	T05G5.9		Ran-binding protein 2-like 4/yeast: ER-Golgi/SNARE assembly	++
VT	W02A11.2		ESCRT-II complex subunit, Yeast: vacuolar protein sorting protein 25	+

U: Unknown, RSP: RNA synthesis and processing, PT: Protein Transport, PS: Protein synthesis, PF: Protein Folding, DR: DNA replication, ECM: Extracellular matrix, CS: Cytoskeleton, EM: Energy metabolism, GM: Glucose Metabolism, SG: Signaling, VT: vesicle transport, ET: electron transport. +: up to a two-fold increase, ++: more than a two-fold increase in the amount of inclusions.

The modifier genes function in a variety of biological processes, some of which have previously been suggested to be involved in Parkinson's disease, such as vesicular transport and lipid metabolism. Lipid metabolism, lipid membranes, and vesicle-mediated transport have previously been linked to α-synuclein pathology in a yeast model [12],[13]. In Parkinson's patients, lipids and membrane material have been found to be directly associated with α-synuclein in lipid droplets and Lewy bodies [11], [14]–[16].

**Figure 5:**
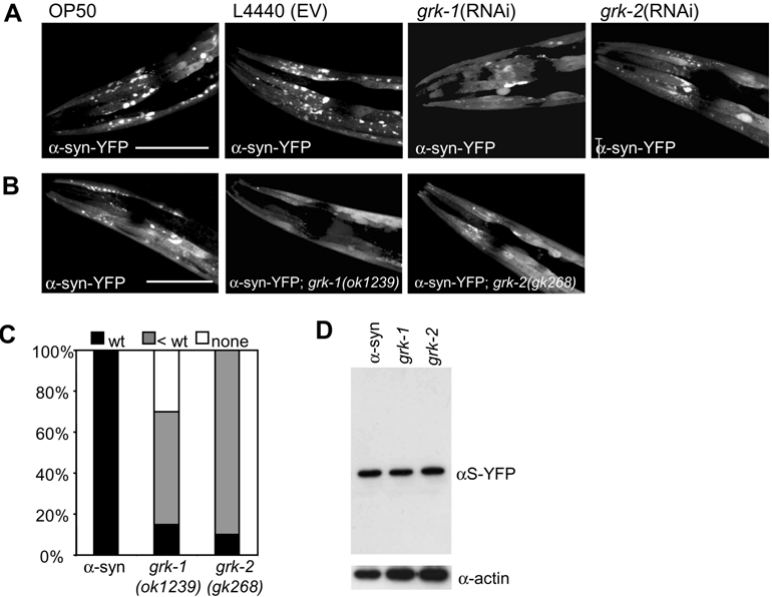
RNAi and Deletion of *grk-1* and *grk-2* Decreases Inclusion Formation. (A) Confocal images showing head region of α-synuclein-YFP transgenic animals fed on OP50 bacteria, bacteria containing empty RNAi vector (L4440), and expressing double stranded RNA targeting *grk-1* and *grk-2*. (B) Confocal images of α-synuclein-YFP *C. elegans,* in wild type background, *grk-1(ok1239)* and *grk-2(gk268)* background. Scale bar represents 25 μm. (C) Quantification of the number of worms within the population (n = 20) with the same amount (wt), fewer (<wt) or no inclusion (none). (D) Western blot analysis of protein extracts from 20 staged transgenic *C. elegans* with a *grk-1* or *grk-2* genetic deletion showing similar levels of fusion protein expressed and actin (loading control).

Although we did identify a *C. elegans* ortholog of the recently discovered modifier of neuronal alpha-synuclein toxicity SIRT2, we did not pick up two other modifiers of neuronal alpha-synuclein pathology: the G-protein coupled receptor kinase 2 and the molecular chaperone Hsp70 [17]–[19]. In *Drosophila,* overexpression of G-protein coupled receptor kinase 2 (Gprk2) increases neuronal toxicity of α-synuclein [18]. In addition, expression of an S129A mutant of α-synuclein, which cannot be phosphorylated by Grpk2, is less toxic, while forming more aggregates [18]. Based on these observations, one might expect that knockdown of Gprk2 would result in an increase in the amount of inclusions as well. Two orthologs of the Gprk2 gene are present in *C. elegans*, *grk-1* and *grk-2*. We tested the effect of knockdown of these two genes by RNAi on the formation of inclusions. Unexpectedly, RNAi knockdown of *grk-1* or *grk-2*, and not RNAi of random tyrosine or serine kinases, resulted in a decrease in the amount of inclusions (Figure 5A and data not shown), indicating that we could not have recovered these genes in our screen for more inclusions. There was no obvious difference in the level of α-synuclein-YFP expression at day 5 after synchronization, indicating that formation of the inclusions themselves is affected (Figure 5D). The effect of knockdown of *grk-1* and *grk-2* by RNAi was confirmed by crossing in deletion alleles of both genes, *grk-1*(ok1239) and *grk-2*(gk268), into the α-synuclein-YFP strain (Figure 5B). Note that an RNAi screen for a reduction in the amount of inclusions yielded only one other kinase, which supports the idea that GRKs act specifically (data not shown). In all, *C. elegans* orthologs of Gprk2 act as modifiers of α-synuclein inclusion formation. Knockdown of Gprk2 in *Drosophila* or overexpression of *grk-1 or grk-2* in *C. elegans* will be required to establish whether their specific role in alpha-synuclein pathology is comparable between the two species.

**Figure 6:**
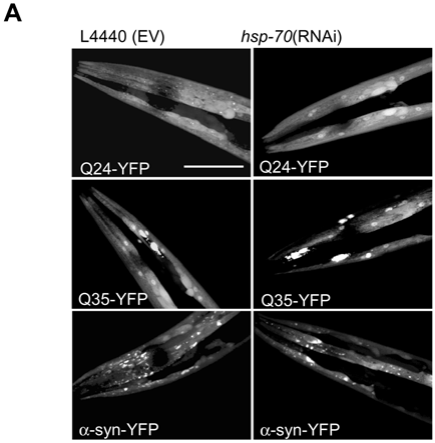
RNAi of *hsp-70* Does Not Affect Inclusion Formation. (A) Confocal images showing Q24-YFP, Q35-YFP and α-synuclein-YFP transgenic animals fed on bacteria containing empty RNAi vector (L4440), and expressing double stranded RNA targeting *hsp-70*.

While Gprk2 overexpression increases toxicity of α-synuclein in *Drosophila*, Hsp70 overexpression has been shown to decrease its toxicity [19]. Because we did not recover Hsp70 as a modifier in our screen, we tested RNAi for the Hsp70 (C12C8.1) gene independently. Knockdown by RNAi of Hsp70 did not obviously increase the amount of inclusions, whereas the same RNAi clone did increase the amount of inclusions in a polyglutamine worm (Figure 6). This result is consistent with the findings in *Drosophila*–that the decreased toxicity in the presence of Hsp70 is independent of a change in the amount of alpha-synuclein inclusions [19]. Apart from a direct role in the detoxification of misfolded monomers or oligomers of alpha-synuclein, Hsp70 in *Drosophila* neurons may also act indirectly, for example as an inhibitor of apoptosis, which may be independent of aggregation [20].

**Figure 7:**
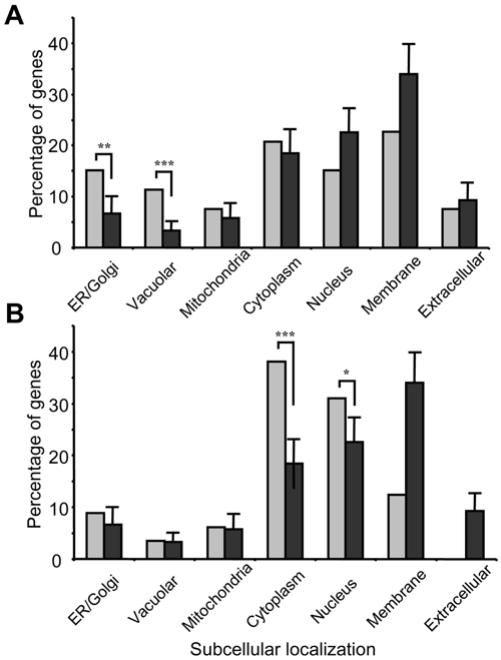
Subcellular Distribution α-Synuclein Modifiers. (A) and (B) bar graphs showing the percentage of genes within each subcellular location class for the genes identified in the α-synuclein modifier screen (A), the polyglutamine modifier screen (B) versus random lists of genes. Random genes were obtained from UniProt (Universal Protein Resource). The subcellular quantities were calculated by adding up the subcellular annotation for 20 sets of 55 randomly chosen proteins (black bars). Values shown are average (n = 20, error bars indicate standard deviation). (A) Overrepresentation of genes in ER/Golgi class and vesicular class in the α-synuclein screen (P-values for the differences between the number of observed genes in ER/Golgi and vesicular class and the number expected: *P*<0.005 and *P*<0.001). (B) Overrepresentation of genes in cytoplasm and nucleus in the polyglutamine screen (P-values for the differences between the percentage of observed genes in cytoplasm and nucleus and the number expected: *P*<0.001 and *P*<0.02).

To further characterize the inclusion mechanism, we classified the complete set of identified modifiers according to seven subcellular locations as annotated by the UniProt entries of the human orthologs (see Table S1) and compared this classification with that of sets of random genes (Figure 7A). This analysis revealed that the number of genes that function in the ER-Golgi and vacuolar compartment are more than 2-fold higher (p<0.005) and more than 3-fold higher (p<0.001), respectively, than would be expected by chance (Figure 7A).

A number of these endomembrane-localized genes are involved in vesicle-mediated trafficking, protein quality control, and detoxification of the ER (Table 2). Of particular interest is the cytochrome P450 gene (*cyp-34A7*), whose closest human ortholog inactivates neurotoxic compounds that are known to increase the risk of developing Parkinson's disease in humans, such as organophosphate insecticides and paraquat [16], [21]–[23].

**Table 2:** Classification of Suppressors with Human Orthologs Functioning in the ER/Golgi and Vesicular Compartments.

Cellular process	Genes	Human ortholog	Molecular process*	Localization
**Vesicle Trafficking**	*W02A11.2*	VPS-associated protein 25	*Component of the ESCRT-II complex, sorting*	Vesicular
	*M151.3*	Girdin	*G{alpha}-interacting vesicle-associated protein*	ER/Golgi
	*hgrs-1*	HGF-regulated tyrosine kinase substrate	*Involved in trafficking and sorting*	Endosomal
	*T05G5.9*	GRIP and coiled-coil domain-containing protein 2	*ER-to-Golgi SNARE complex assembly*	Golgi
**Protein Quality Control**	*F49B2.6*	Leucyl-Cystinyl aminopeptidase	*Degrades peptide hormones*	Vesicular
	*F21F3.3*	Methyltransferase	*Membrane attachment (folding-localization)*	ER
	*ugt-34*	UDP-glucuronosyltransferase 1-8 precursor, UGT	*Carbohydrate transferase (folding)*	ER
	*spp-10*	Proactivator polypeptide precursor	*Lipid transport, glycosphingolipid metabolism*	Lysosomal
	*vha-15*	Vacuolar ATP synthase subunit H	*Vacuolar acidification*	Vesicular
	*R151.6*	Derlin-2	*ER homeostasis and translocation*	ER
**Other**	*cyp-34A7*	Cytochrome P450 2B6	*Detoxification in ER*	ER
	*tag-160*	Isoform 1 of LAG1 longevity assurance homolog 1	*ceramide/glycosphingolipid metabolism*	ER

*: Genes are annotated to the endomembrane system by their UniProt IDs, see Table S1

We also identified several molecular regulators of lifespan as modifiers of inclusion formation, such as *lagr-1*, a sphingolipid synthase, and *sir-2.1*, the *C. elegans* homolog of Sirtuin1, both of which we confirmed by crossing the deletion strain for both genes, *lagr-1(gk331)* and *sir-2.1(ok434)*, into the α-synuclein strain (Figure 4 A–4D) [24]. Deletion of *sir-2.1* or *lagr-1* both result in an increase of approximately 35% in the number of α-synuclein inclusions in nine-day old adult worms (Figure 4 A-4D). The homolog of *lagr-1* in yeast, LAG1, regulates its lifespan and is homologous to LASS2 in humans. Sirtuin-1 is an NAD+-dependent protein deacetylase, which regulates lifespan in yeast, fruit fly and *C. elegans* [25]–[28]. In *C. elegans*, overexpression of sir-2.1, the homolog of human sirtuins 1 to 3, extends its lifespan by up to 50%, probably by inhibiting expression of ER-stress genes [25],[26]. These findings suggest a link between the molecular mechanism of aging and α-synuclein pathology.

In all, our screen recovered genes and processes that have previously been identified in other cell or animal models as modifiers of neuronal α-synuclein toxicity, such as *sir-2.1* and genes involved in vesicle trafficking [12],[17],[29]. Based on these findings, we expect other genes from our screen to provide insight into neuronal α-synuclein pathology as well. Future studies in neurons will resolve which of these gene processes are implicated in neuronal α-synuclein pathology.

In a screen performed previously for modifiers of polyglutamine aggregation in *C. elegans*, we found a large variety of proteasomal genes and chaperones [6]. These genes are typically found to be involved in protein misfolding and aggregation and are suggested to be generally involved in protein-misfolding diseases. Surprisingly, very few of such genes were found in the α- synuclein screen. Only a single gene found to increase α-synuclein inclusion overlapped with the screen for polyglutamine aggregation we performed in *C. elegans*. Perhaps even more strikingly, the modifiers of polyglutamine aggregation that we had identified previously located primarily to cytosol and nucleus (Figure 7B) [6]. This suggests that different cellular processes are involved in preventing the development of inclusions in these protein-misfolding disorders. In conclusion, we have developed a transgenic *C. elegans* model showing α-synuclein inclusions that share important characteristics of inclusions in Parkinson's disease. The formation of inclusions shows a strong age-dependency and clearly distinct, consecutive phases of progressive aggregation. Our transgenic worms therefore provide a versatile model for the characterization of the molecular steps preceding pathologic inclusion formation. In a global RNAi screen we identified modulators of α-synuclein aggregation that are distinct from those in other protein-aggregation disorders. Most importantly, our list of suppressors of age-dependent inclusion formation reveals a clear link between α-synuclein inclusion formation and cellular aging, most probably via an endomembrane-related mechanism. Our results provide an important guide for further elucidation of the pathogenic mechanisms and genetic susceptibility factors in human Parkinson's disease and other α-synuclein related disorders.

## Materials and Methods

### Constructs

To create P(unc-54)α-synuclein: YFP (pRP2386) α-synuclein was amplified from cDNA (provided by S. Lindquist, MIT, MA) by PCR using primers adding a *KpnI* and a *AgeI* site, respectively (3′-agcgtcgacggtaccgcgatggatgtattcatgaaagg-5′, 3′- gactacgaacctgaagcctccaccggt cgccacctc-5′), to the α-synuclein gene, which was then cloned into the pPD30.38-YFP expression vector.

### Media and Strains

Standard conditions were used for *C. elegans* propagation at 20°C. Worms were synchronized by hypochlorite bleaching, hatched overnight and were subsequently cultured on NGM plates with OP50. On day 3 after synchronization, worms were placed on NGM plates containing 5- Fluoro-2′deoxy-uridine (FUDR) to prevent eggs from hatching. The following strains were used: NL5901 (pkIs2386 [α-synuclein::YFP unc-119(+)]),VC199(ok434), OW8 (sir- 2.1(ok434)IV; pkIs2386[α-synuclein::YFP unc-119(+)]), VC765(gk331), OW13 (grk-1(ok1239)X; pkIs2386[α-synuclein::YFP unc-119(+)]),OW15 (grk-2(gk268)III; pkIs2386[α-synuclein::YFP unc-119(+)]),OW6 (ymel-1(tm1920)III; pkIs2386[α-synuclein::YFP unc-119(+)]), OW101 (lagr-1(gk331)I; pkIs2386[α-synuclein::YFP unc-119(+)]), AM134 (rmIs126[P(unc-54)Q0::YFP]), and AM141 (rmIs133[P(unc-54) Q40::YFP]) [7]. The α-synuclein YFP fusion is driven by an *unc-54*, muscle specific, promoter to obtain expression in the body-wall muscle cells. Muscle expression was chosen to allow for efficient RNAi and for visual detection of inclusions [6]–[8].

### Creation of Transgenic Strains

Transgenic *C. elegans* were generated using standard ballistic transformation by co-bombarding unc-119 rescue vector (pRP2512) and the α-synuclein-YFP expression construct into unc- 119(*ed3*) nematodes [30].

### Immunoblotting Analysis

Nematode protein extracts were prepared from whole animal frozen pellets in PBS containing proteinase inhibitors (Roche, Indianapolis, USA) by using Fastprep24 (MP, Solon, USA). Samples were boiled 5 min with sample buffer containing SDS and DTT, and separated on 10% SDS/PAGE. Proteins were transferred to PVDF membranes. Antibody binding was visualized by binding of horse-radish peroxidase-coupled secondary antibody and chemiluminescence (ECL Lumi-Light, Roche, Germany). Antibodies used were α-synuclein (1:3,000; LB509, Zymed, San Francisco, USA), anti-actin (1:8,000; Clone C4, ICN, Aurora, OH, USA) and anti-GFP polyclonal serum [31]. Goat-anti-mouse and goat-anti-rabbit IgG-HRP (1:5,000; BioRad, Hercules, CA, USA) were used as secondary antibodies.

### CLSM and Fluorescence Recovery after Photo Bleaching (FRAP)

Transgenic worms were mounted on 2% agarose pads containing 40 mM NaN3 as anaesthetic on glass microscope slides. Images were captured using a Leica DMIRE2 confocal microscope with an x40 oil immersion lens (HCX PL APO CS). Images shown are 2D maximal projections of zseries. Immobilized animals were subjected to FRAP analysis with minor modifications as described [20]. An area of ∼4 µm2 was bleached for 5 s (five iterations at 100% intensity), after which images were collected (at low intensity). Relative fluorescence intensities (RFI) were calculated as described by using the RFI = (*Net*/*N*1*t*)/(*Ne*0/*N*10) equation [10]. For quantification of the number and motility of inclusions (Figure 1 y and z) all foci larger than 2 µm2 between the nose and pharyngeal bulb were analyzed by FRAP. Measurements on inclusions were performed using ImageJ software [26]. Statistical significance was determined using *t* tests.

### RNAi Screen

Synchronized larvae were grown in liquid culture in 96-well plates, on bacterial strains from the Ahringer bacterial RNAi library as described [6],[32]. Worms grown on the different RNAi clones were visually selected for increased inclusion formation four and five days after bleaching. Bacterial clones, that exhibited increased inclusion formation, were confirmed in an independent experiment in duplicate in liquid culture. RNAi clones confirmed to increase inclusion formation were then fed to α-synuclein-YFP transgenic *C. elegans* on NGM plates for reconfirmation. All gene targets of the positive-RNAi foods were verified by sequencing of the insert of the RNAi plasmids.

### Bioinformatics and Statistical Analysis

Subcellular location data were tested for overrepresentation using expected relative frequencies based on a Poisson distribution (e-μμY/Y!), where Y is the observed percentage of genes observed for a given class (Y) and μ is the expected (mean) percentage of this class among all annotated genes. The observed results were compared to a random list of 55 genes (n = 20) created from random UniProt entries with annotated subcellular locations. Genes functioning in more than one location were assigned to both locations.

## Supporting Information

Table S1 Human orthologs.
(0.08 MB XLS)
